# How non-specific low back pain affects gait kinematics: a systematic review and meta-analysis

**DOI:** 10.3389/fpain.2025.1693068

**Published:** 2025-11-20

**Authors:** Fulvio Dal Farra, Nicola Francesco Lopomo, Matteo Fascia, Emilia Scalona, Serena Cerfoglio, Veronica Cimolin

**Affiliations:** 1 Department of Information Engineering, University of Brescia, Brescia, Italy; 2 Department of Design, Politecnico di Milano, Milan, Italy; 3 Medical and Surgical Specialties, Radiological Sciences and PublicHealth (DSMC), University of Brescia, Brescia, Italy; 4 Department of Electronics, Information and Bioengineering, Politecnico di Milano, Milano, Italy; 5 IRCCS Istituto Auxologico Italiano, San Giuseppe Hospital, Piancavallo, Italy

**Keywords:** low back pain, kinematics, gait analysis, gait speed, sensors, walking, IMU, optoelectronic

## Abstract

**Background:**

Non-specific low back pain (NS-LBP) is a is a highly prevalent musculoskeletal condition, with an estimated 619 million prevalent cases worldwide in 2020. Alterations in spinal and lower limb dynamics are considered as potential factors directly involved in this condition, thus we carried out a systematic review to summarize the evidence regarding walking kinematics in NS-LBP.

**Methods:**

The reporting of this review followed the “2020 Preferred Reporting Items for Systematic Reviews and Meta-Analysis” (PRISMA 2020 checklist) and the protocol was preliminary registered in PROSPERO (ID: CRD42023431380). A search strategy was implemented in Medline, Embase, Scopus, Web of Science, and IEEE Xplore databases, up to March 2024. Inclusion criteria were: any analytical observational research instrumentally assessing the trunk and lower limbs kinematics of spontaneous walking in NS-LBP, in a comparison with healthy people. Study selection and data extraction were performed by two blinded reviewers, the methodological quality was evaluated by the Joanna Briggs Institute (JBI) Critical Appraisal Checklist and the quality of the evidence was rated through GRADE criteria.

**Results:**

Overall, a total of 19 cross-sectional studies were included in this review and none of those was found without methodological issues. The meta-analysis showed a lower gait velocity [−15.42 (−22.78, −8.06) cm/s; *p* ≤ 0.0001], a lower cadence [−9.85 (−18.72, −0.99) steps/min; *p* = 0.03] and a lower step length [−6.30 (−11.83; −0.77) cm; *p* = 0.03] in NS-LBP. Regarding motion analysis, a few authors observed a less and asymmetrical motion of the lower spine in the frontal and in the transverse plane.

**Conclusion:**

There is very-low quality evidence that gait speed, cadence and step length are reduced in patients with NS-LBP. There is proof of a movement reduction in the lower lumbar spine and in the pelvis, both in the transverse and in the frontal plane. No differences in the lower limb kinematics was consistent over the studies.

**Systematic Review Registration:**

https://www.crd.york.ac.uk/prospero/, identifier CRD42023431380.

## 1 Introduction

Low back pain (LBP) is a highly prevalent musculoskeletal condition, with an estimated 619 million prevalent cases worldwide in 2020 (1). Beyond its clinical impact, LBP represents a major social and economic burden, with total costs reaching billions of dollars annually in high-income countries (2) However, in a vast majority of the cases (57%–89%), no specific etiology is identifiable so the expression “non-specific LBP” (NS-LBP) is commonly used (3).

In this context, several underlying components have been hypothesized to contribute to LBP development, including age, gender, socio-demographic features, psychological characteristics, lifestyle, and mechanical issues. Therefore, LBP etiology can be properly defined as multifactorial (4). Mechanical factors, are suggested to be related to repetitive and prolonged stresses on the spine. These factors can be associated with posture and movement issues, and seem to be related to the development and persistence of the pain (5). More in detail, perturbations in spinal and lower limb dynamics have been considered as potential factors directly involved in NS-LBP, including joint rigidity, muscle stiffness or weakness, and poor neuromuscular function (e.g., altered timing of activation, incoordination); in fact, all these factors could lead to asymmetrical or abnormal mechanical loading of the lumbar spine (6–8).

Since walking represents one of the human activities that is frequently and routinely repeated throughout the whole day, it can be affected and contribute to pain, activity limitations, and disability in subjects presenting NS-LBP (9). For this reason, a quantitative and reliable evaluation of walking is of paramount importance when assessing subjects with NS-LBP. According to the main findings present in scientific literature, there is proof that the speed of gait in NS-LBP is, in fact, lower than in healthy people, whereas there is no consensus regarding other spatio-temporal parameters (10, 11). In the same way, there are conflicting results concerning how NS-LBP can affect the movement observed in different planes of motion; several authors found in fact that NS-LBP subjects showed a reduced range of motion in the axial plane, while others reported wider ranges of spinal and pelvic rotations (9–13).

Despite the large number of studies carried out over the years, to the best of our knowledge, no systematic review exists on this topic, so far; this lack presents certain implications since the absence of shared evidence and/or common guidelines prevents investigators to address future research and clinicians to develop tailored rehabilitative treatments for specific subgroups of NS-LBP subjects (14).

Therefore, we hypothesized that there is a potential associationbetween NS-LBP and modifications of the spinal and lower limb kinematics during walking when compared to healthy people. In this frame we carried out a systematic review focused to provide a structured summary of the actual evidence on this specific topic, specifically addressing the use of quantitative and instrumental assessing methods.

## 2 Methods

The reporting of this systematic review followed the “2020 Preferred Reporting Items for Systematic Reviews and Meta-Analysis” (PRISMA 2020 checklist) (15). A “PECO” strategy was used to state the research question (P: non-specific low back pain; E: exposure to changes in kinematics during walking; C: kinematics in healthy people; O: each kinematic indicator measured through instrumental assessment) (16). The protocol was regularly approved and published in the international prospective register of systematic reviews (PROSPERO, https://www.crd.york.ac.uk/prospero/, registration ID: CRD42023431380).

### 2.1 Search strategy and eligibility criteria

A systematic search was conducted to establish if kinematics is altered in NS-LBP subjects. Medline (PubMed), Embase, Scopus, Web of Science, and IEEE Xplore databases were consulted up to January 2024 and monthly updates were carried out until January 2025. In addition, we performed cross-referencing to retrieve any possible missing studies, and grey literature was also considered through Google web searching and Google Semantic Scholar. Different search terms and keywords were used, such as “low back pain”, “spinal pain”, “backache”, “kinematics”, “biomechanics”, “gait”, “walking”, “locomotor”, “sensor” and “optoelectronics”. These words were combined differently according to database search criteria. Inclusion criteria for this review were the following: any kind of analytical observation research including case-control, cohort and cross-sectional studies, assessing the trunk and lower limbs kinematics of spontaneous walking (preferred speed) in NS-LBP subjects, and comparing it with that of healthy people. Randomized controlled trials were excluded, as the purpose of this review was not to assess the effectiveness of interventions, but to synthetize evidence on kinematic characteristics in NS-LBP.

We included only studies that evaluated kinematics by using instrumental assessing methods, including—for instance—marker-based optoelectronic systems and wearable sensors. Further considered criteria were adult population (18–70 years old), and English as the main language to ensure homogeneity of data extraction and interpretation, and to allow reproducibility by the scientific community. As for the exclusion criteria, we did not consider studies where walking was assessed on the treadmill or studies dealing with subjects affected by other conditions which could affect the walking performance (e.g., neurological, orthopedic, and rheumatic comorbidities). Due to the intrinsic variability of gait assessment, no restriction in terms of protocol was applied, except for the spontaneity of walking (preferred speed) which had to be present.

### 2.2 Study selection and data collection

**Figure 1:**
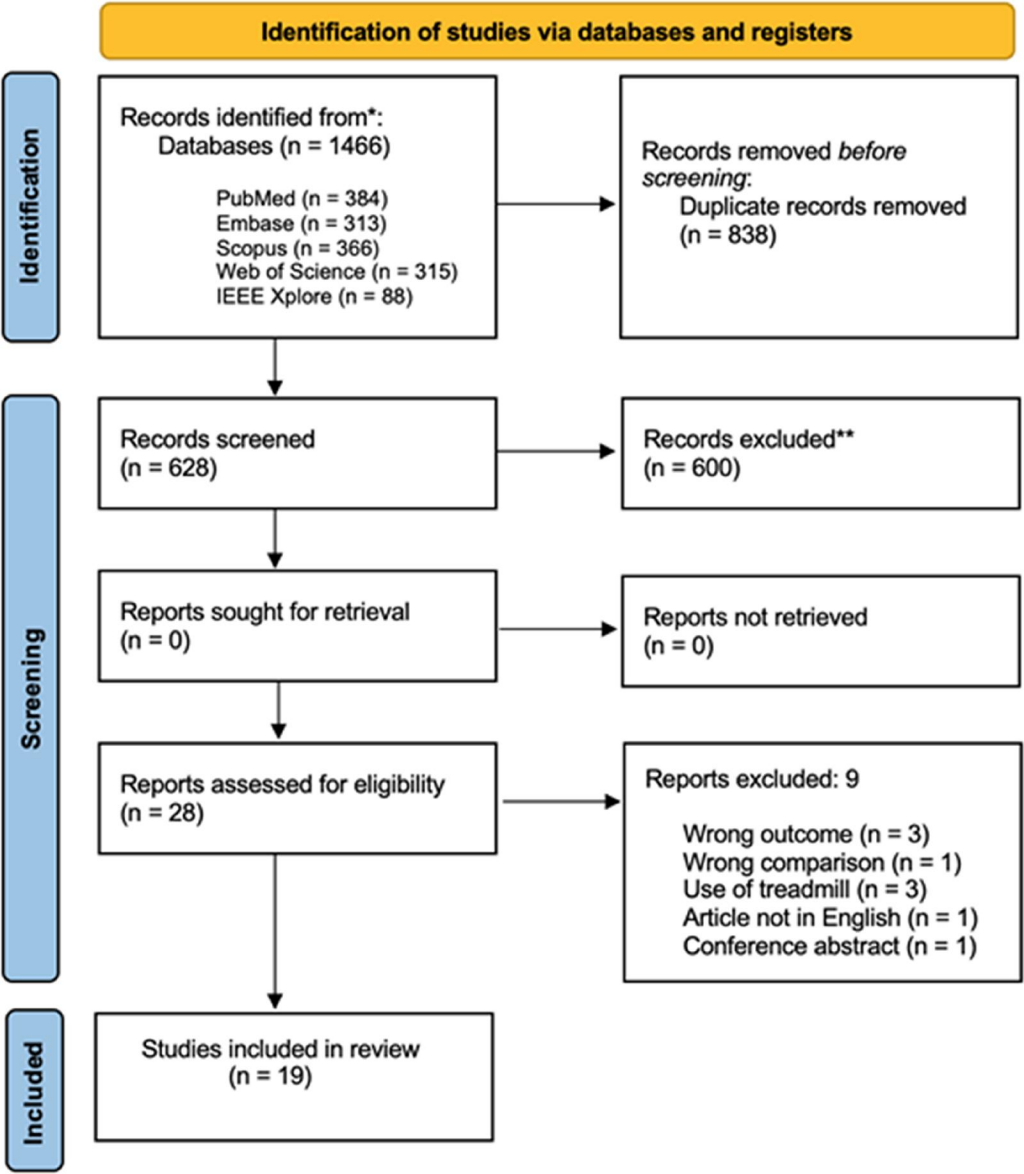
Flow diagram based on PRISMA statement (https://www.prisma-statement.org).

All the records obtained from the search process were managed using “Rayyan, Intelligent Systematic Review” (https://www.rayyan.ai) (17). Title, abstract, and full texts were screened independently by two blinded reviewers (FDF, MF) to identify eligible studies. Any discrepancy was resolved through a consensus with another reviewer (VC). At the time the first screening procedure was conducted (2023), this software had not yet implemented any artificial intelligence-based based features. Details of the study selection stage are reported in the PRISMA 2020 flow diagram (Figure 1). Main characteristics and the major findings of the included studies were extracted in a standardized form and summarized in a table (Table 1) reporting first author name and year of publication, study design, main objective, outcomes, sample size, the technology employed in the gait assessment and the main results obtained according to the aim of this review. The data extraction form was developed and approved by all the authors involved in this study, and a 6-h consensus training was implemented before starting. Once again, the same reviewers independently screened the included studies and resolved any disagreement through a discussion.

**Table 1:** Characteristics of the included studies.

Author, year (country)	Study design	Objective	Sample	Outcomes	Technology	Results
Al-Obaidi et al. (21) (Kuwait)	Cross-sectional study	To examine the influence of of pain, pain related fear and disability beliefs on the gait characteristics CLBP patients at preferred and fast performance	N: 55 31 CLBP and 24 healthy subjects (28 F, 27 M, age: 37.7 ± 7.54) Mean 0–100 VAS pain score for CLBP group: 31.5 ± 15.7	Spatiotemporal parameters*: step time (s), step length (cm), single support (%), velocity (cm/s), cadence (steps/min)*	Sensitive computerized mat (GAITRite system, CIR Systems Inc., Clifton, New Jersey, USA)	Difference between healthy and CLBP subjects both at the preferred and the fast performance for step length, single support (right side) and velocity (*p* ≤ 0.05). Difference only in women for cadence at preferred speed (*p* ≤ 0.05).
Amir Rashedi Bonab et al. (22) (Turkey)	Cross-sectional study	To assess spatiotemporal gait parameters in patients with lumbar disc herniation (LDH) and chronic mechanical low back pain (CMLBP), and compare with healthy control group.	N: 70 25 LDH, 25 CLBP and 20 healthy subjects (age: 25–65) Mean 0–10 VAS pain score for CLBP group: 5.7	Spatiotemporal parameters: *step time (s), gait cycle (s), double stance time (s), swing time (s), step length (cm), gait cycle length (cm),velocity (mm/s), cadence (steps/min)*	WIN-TRACK (Medicapteurs, Balma, France) gait analysis platform system	The spatiotemporal gait parameters were significantly decreased in LDH and CMLBP groups than the healthy control group, particularly in LDH groups (*p* ≤ 0.001).
Christe et al. (30), (Switzerland)	Cross-sectional	To provide a comprehensive comparison of spinal kinematics during gait between CLBP patients and asymptomatic controls	N: 21 10 CLBP and 11 healthy subjects (9 F, 12 M, age: 37.7 ± 6.3) Mean 0–10 NRS pain score for CLBP group: 3.7 ± 2 Mean ODI score for CLBP group: 24.2 ± 9.8	3D-angles at: *lower lumbar joints (LLS), upper lumbar joints (ULS), lower thoracic joints (LTS), upper thoracic joints (UTS)*	Optoelectronic system (VICON, Oxford Metrics, UK)	CLBP patients had significantly less frontal-plane motion at the LLS joint and more asymmetrical transverse-plane motion at the LTS joint than controls (*p* ≤ 0.05)
Cimolin et al. (31), (Italy)	Cross-sectional study	To quantify the gait pattern of obese subjects with and without LBP and normal-mass controls by using gait analysis	N: 28 8 obese CLBP, 10 obese and 10 healthy subjects (28 F age: 40.5 ± 10.1)	Spatiotemporal parameters: *stance (%)* *double stance phase (%);* *normalized mean velocity (*1*/s);* *normalized step length;* *cadence (steps/min)* Angles peak: *ankle dorsiflexion during stance and swing phase, knee flexion during swing;* *- ankle plantarflexion in stance and of knee extension during gait cycle.* ROM *Hip on the coronal and sagittal plane; the knee on the sagittal plane; the ankle on the sagittal plane during stance.*	Optoelectronic system with 6 cameras (460 VICON, Oxford Metrics Ltd., Oxford, UK)	Obese CLBP showed longer stance duration and shorter step length when compared to obese and healthy subjects. Obese CLBP had a lower pelvis and hip ROM on the frontal plane, a low knee flexion in the swing phase and knee ROM, a low ankle dorsiflexion in stance and swing as compared to obese (*p* ≤ 0.05)
Crosbie et al. (23), (Brazil)	Observational cohort study	To compare temporal and spatial coordination of rotations in the transverse and frontal planes of the lower thoracic and lumbar spinal regions during walking at preferred and fast speeds in 2 groups of participants with and without a history of recurrent LBP	N: 38 19 recurrent LBP and 19 healthy subjects (25 F, 13 M; age: 31.3 ± 9.35) Mean 0–10 VAS pain score for CLBP group: 2.8 ± 2.2 Mean RMDQ score for CLBP group: 4.2 (0–19)	*Peak to peak of lower thoracic, lumbar, pelvis and hip* Coordination within and between spinal segments *(lower thoracic and lumbar)* for side flexion and axial rotation *Harmonicity index (IH) between spinal segments (lower thoracic, lumbar and plevis)*	Multisensor, 6-df electromagnetic tracking device (Motion Star Wireless 2 system; Ascension Technology Corporation, Burlington, VT)	ROM: the only between-group difference was a *smaller pelvic side* *flexion* at preferred speed in persons with LBP (*p* ≤ 0.05) Coordination between segments: *phase lag* for axial rotation was significantly less in subjects with LBP than controls at a preferred speed (*p* ≤ 0.01) Coordination within segments: the lower thoracic region displayed greater coherence in subjects with LBP than in controls (*p* < 0.001) Harmonicity: differences between groups for axial rotation in the lower thoracic and lumbar regions and for side flexion in the lumbar region at both walking speeds, with subjects with LBP displaying greater IH values (*p* ≤ 0.05)
Jiménez-Del-Barrio et al. (32), (Spain)	Cross-sectional study	To compare pelvis, hip and knee kinematics during gait between LBP and asymptomatic subjects.	N: 40 20 NS-LBP and 20 healthy subjects (29 F, 11 M, age: 24.6 ± 5.21) Mean 0–10 VAS pain score for NS-LBP group: 5.68 ± 2.23 Mean ODI score for CLBP group: 25.15 ± 8.03	Spatiotemporal parameters: *Step length (cm), stance(s) and swing phase (s)* ROM *Hip and knee in the sagittal and frontal planes*	2D Kinovea Software v. 8.15	Increase in pelvic tilt (*p* < 0.01), valgus angle (*p* < 0.01), and a significant decrease in hip extension (*p* < 0.01) in the NS-LBP group compared to the healthy subjects.
Ebrahimi et al. (33), (Iran)	Cross-sectional study	To compare the trunk-pelvis and lower extremities sagittal plane inter-segmental coordination and variability during walking in persons with and without CLBP	N: 20 10 CLBP and 10 healthy subjects (10 F, 10 M, age: 29.6 ± 5.64) 0–10 VAS pain score for LBP group: [range: 4–6] ODI score for CLBP group: [range: 21–60]	Coordination *Trunk-pelvis and bilateral pelvis-thigh coordination pattern.* *Thigh-shank and shank-foot coordination pattern*. *Mean absolute relative phase (MARP) Deviation phase (DP)*	8-camera motion analysis system (Proreflex, Qualisys Track Manager Ltd., Gothenburg, Sweden) at a sampling rate of 100 Hz	CLBP group demonstrated less variable right or left pelvis-thigh coordination pattern (*p* < 0.05). The left thigh-shank and left shank-foot MARP values in the CLBP group were more in-phase than left MARP values in the non-CLBP control group during the swing phase (*p* < 0.05).
Farahpour et al. (34), (Iran)	Cross-sectional study	To investigate whether excessive feet pronation alters the joints’ kinematics, kinetics and the activity of involved muscles during gait in low back pain patients	N: 45 15 LBP + pronated feet, 15 only with pronated feet + 15 healthy subjects (age: 25.9 ± 2.8)	Angles: *peak joint angles at hip, knee and ankle joints in stance phase of walking.*	Vicon MX motion analysis system including 4 T-series cameras (100 Hz) was used to quantify gait kinematics. A Kistler force plate (Kistler AG, Winterthur, Switzerland) was used to record the GRF components at 1,000 Hz.	Significant difference in peak angles between LBP and other groups in ankle inversion and eversion, knee flexion and external rotation (*p* < 0.05).
Gombatto et al. (23), (United States)	Cross-sectional study	To determine differences in magnitude and symmetry of the upper and lower lumbar spine kinematics between people with LBP and people without LBP during walking.	N: 36 18 LBP and 18 healthy subjects (21 F, 15 M, age: 27.8 ± 12.7) Mean 0–10 NRS pain score for LBP group: 2.1 ± 1.9 Mean modified ODI score for LBP group: 18 ± 12.07	Spatiotemporal parameters *Gait speed (m/s)* *Cycle time (s)* *Stride length (m)* *Step length (m)* Maximum and minimum spine segment angles on axial, coronal and sagittal planed for *upper* and *lower lumbar spine.*	A 9-camera, 3-D movement analysis system (Vicon, Inc.)	People with LBP displayed significantly less overall lumbar rotation than controls (*P* < 0.05).
Lee and Sung (24), (United States)	Cross-sectional study	To compare gait parameters as well as combined limb motions between subjects with and without LBP	N: 41 22 LBP and 19 healthy subjects (18 F, 23 M, age: 28.2 ± 11) Mean ODI score for LBP group: 21.18 ± 5.6	Spatiotemporal parameters *Cadence (steps/min)* *Speed (cm/s)* *Stride length (cm)* *Step width (cm)* *Stance (%)* *Swing (%)* Gait similarity: *Kinematic similarity index (KSI)* in hips, knees and ankles	Motion Analysis System (Motion Analysis Corporation, Rohnert Park, CA, USA) with 6 infrared cameras, 2 force platforms (AMTI OR6–7, Advanced Mechanical Technology, Inc.,Watertown, MA, USA) sampling at 120 Hz. The ground reaction forces were recorded at 1,000 Hz	The KSI of the control group demonstrated higher similarities in the swing (*p* = 0.001) and stance (*p* = 0.001) phases compared to the LBP group. The KSI for the whole gait cycle was significantly different between the groups (*p* = 0.001), especially in the midstance and swing phases.
MacRae et al. (25), (United Kingdom)	Cross-sectional study	To determine whether participants with CLBP have similar or different gait parameters, when compared with age-matched and gender-matched asymptomatic participants	N: 32 12 LBP and 12 healthy subjects (16 F, 16 M, age: 37 ± 10.6) Mean 0–10 VAS pain score for LBP group: 5.9 ± 1.5	Spatiotemporal parameters *Speed (m/s)* *Cadence (steps per min.)* *Stride length (m)* ROM *Max, min and total Hip range of motion in the sagittal plane*	Vicon's Nexus (V.1.8.1) motion capture software (Vicon Motions Systems, Oxford, UK), with 7 cameras, capturing retroreflective markers in 3D space at a rate of 120 Hz.	No differences were observed between groups for spatiotemporal parameters, maximum, minimum and total ranges of hip movement.
Müller et al. (9), (Germany)	Cross-sectional study	To investigate whether CLBP causes modifications in lower limb and trunk movements	N: 22 11 CLBP and 11 healthy subjects (11 F, 11 M, age: 38.3 ± 13)	Spatiotemporal parameters *Speed (m/s)* *Stride length (m)* ROM *Max, min degrees of thorax, pelvis and trunk in the transverse plane.* *Trunk, knee and ankle in the sagittal plane.*	The ground reaction forces were sampled at 2,000Hz by using (9,281B and 9,287BA Kistler, Winterthur, Switzerland) 8 cameras (240 Hz) by a 3D infrared system (MCU 1,000, Qualisys, Gothenburg, Sweden)	During walking, CLBP patients showed a 6.5% reduced preferred walking speed compared to healthy people (*p* < 0.01). As compared to healthy controls, the amplitude of pelvis rotation decreased significantly in CLBP patients (*p* < 0.05), the knee joint angle at touchdown was significantly more extended than in healthy controls (*p* < 0.05).
Mullerpatan et al. (35) (India)	Cross-sectional study	To explore spine, pelvis, and lower-extremity kinematics during gait performance among professional Bharatanatyam dancers with and without LBP	N: 27 7 NS-LBP dancers, 10 healthy dancers and 10 healthy non-dancers (27 F, age: 21.4 ± 1.3)	ROM *Max angles of spine extension, anterior pelvic tilt, pelvic obliquity and rotation, hip flexion, extension, ext. rotation, abduction, knee flexion and extension, ankle flexion and extension*	12 Vicon infrared cameras (Oxford Metrics Group, London, UK). A 3D motion analysis system was used to capture motion at a sampling frequency of 100 Hz	Dancers with NS-LBP exhibited 20% increased spine extension, 35% increased anterior pelvic tilt, 57% reduced pelvic obliquity, 30% reduced pelvic rotation, 40% greater hip flexion, and 56% increased ankle plantar flexion compared to dancers without low-back pain (*p* ≤ 0.05).
Nishi et al. (36) (Japan)	Cross-sectional study	To investigate the impacts of the environment and LBP on the trunk variability and stability of gait in CLBP patients.	N: 40 20 LBP and 20 healthy subjects (17 F, 23 M age: 55.4 ± 10.9) Mean 0–10 NRS pain score for LBP group: 4.4 ± 1.32	Trunk variability *Average standard deviations of the AP and ML trunk accelerations* *Multiscale sample entropy (MSE)*	Wearable tri-axial accelerometer (Axivity AX3, York, UK) on L5 capturing data at 100 Hz at a range of ±8 g.	The trunk variability in the CLBP group was significantly high regardless of the environment in the AP direction and high but affected by the environment in the ML dirtion (*p* < 0.05). The MSE was not affected by CLBP status or by the environment in the AP direction, but was high in the CLBP group and affected by the environment in the ML direction (*p* < 0.05).
Rahimi et al. (26), (Iran)	Cross-sectional study	To understand the movement pattern of lower extremities and its asymmetry during walking in people with CLBP	N: 40 20 CLBP and 20 healthy subjects (20 F and 20 M age: 37.2 ± 6.6)	Spatiotemporal parameters *Speed (m/s)* *Stride length (m)* Motion patterns: *lower limbs on the transverse, sagittal and frontal planes*	7 camera Qualysis Proreflex system was used to obtain the three-dimensional (3D) coordinate kinematic data of the lower limbs.	The CLBP group showed significantly different hip motion pattern in the transverse plane, altered knee and ankle motion pattern in the sagittal plane on the dominant side and different hip motion pattern in the transverse and frontal planes on the non-dominant side in comparison with the control group, over the stance phase (*p* < 0.05). Symmetry: in the CLBP group, hip and knee moved through a significantly different motion patterns in the transverse plane on the dominant side in comparison with the nondominant side (*p* < 0.05).
Rum et al. (27), (Italy)	Cross-sectional study	To assess if CLBP elicit neuromuscular and kinematic changes which are specific to walking	N: 22 11 CLBP and 11 healthy people (14 F, 8 M age: 27 ± 7) Mean 0–10 VAS pain score for CLBP group: 4.6 ± 2.4 Mean ODI score for CLBP group: 12.5 ± 3.4	Spatiotemporal parameters *Speed (m/s)* *Normalised Stride length (m/LL)* *Stride time (s)* *Normalized step length (m)* *Step time (s)* ROM *Thorax, lumbar and pelvis motion in the sagittal, frontal and transverse planes* Variability *Mean standard deviations*	A 7-infrared camera motion capture system (Vicon MX3, Oxford, UK) used to reconstruct the 3D position of 35 retro-reflective markers	CLBP group reported greater transverse ROM of the lumbar segment during walking compared to healthy controls. Greater overall movement variability in the transverse plane was observed in the CLBP group while walking (*p* < 0.05).
Simmonds et al. (28), (USA)	Cross-sectional study	To evaluate the influence of pain distribution on gait characteristics in subjects with LBP during walking at preferred speed	N: 60 20 LBP, 20 referred leg pain (LBP + LP), 20 healthy subjects (36 F, 24 M age: 46.2 ± 10.8) Mean 0–10 VAS pain score for LBP group: 4.4 ± 2.4 Mean RMDQ score for LBP group: 9.5 ± 5.9	Spatiotemporal parameters *Speed (m/s)* *Normalized speed (% body height/s)*	Force platform (Advanced Medical Technology Inc.) (AMTI) Model OR6-7–2,000	Gait velocity was highest in the control group followed by the LBP and LBP + LP group and differed between groups at both walking speeds (*p* < 0.001)
Simonet et al. (37), (Switzerland)	Cross-sectional study	To explore possible CLBP related alterations in lumbar lordosis angle during walking	N: 33 13 NS-LBP and 20 healthy subjects (16 F, 17 M age: 34.7 ± 10.4) Mean 0–10 VAS pain score for LBP group: 2.3 ± 1.1 Mean ODI score for NS-LBP group: 19.5 ± 9.2	ROM *Lumbar lordosis angle (LLA)*	10 infrared cameras (VICON, Oxford, UK; sampling rate: 200 Hz).	Patients with CLBP indicated reduced average LLA during walking and a tendency for lower LLA-ROM during walking compared to healthy people (hypothesis not tested) The largest group differences occurring around 25% and 70% of the walking cycle.
Vickers et al. (29), (USA)	Cross-sectional study	To determine whether awareness of being observed differentially affected gait pattern in people with and without CLBP	N: 55 25 CLBP and 30 healthy subjects (28 F, 27 M) Mean ODI score for CLBP group: 30.8 ± 15.4	Spatiotemporal parameters *Speed (cm/s)* *Cadence (steps per min.)* *Stride length (cm)* *Step width (cm)* *Single support time (% of gait cycle)* *Double support time (% of gait cycle)*	GaitRite (CIR Systems, Inc.; Havertown, PA)	The CLBP group reported slower velocity (6–14.6% slower), shorter step length (3.6–9.2% shorter), and lower cadence (3.2–6.3% lower) (*p* < 0.05).

CLBP, chronic low back pain; LDH, lumbar disc herniation; NRS, numeric rating scale; ODI, oswestry disability index; ROM, range of motion; VAS, visual analogue scale; AP, antero-posterior; ML: medio-lateral.

In case of missing data, investigators of the included studies were contacted via email.

### 2.3 Methodological quality assessment

Considering that all the included studies are attributable to a cross-sectional design (see Results section), the Joanna Briggs Institute (JBI) Critical Appraisal Checklist for Analytical Cross-Sectional Studies was embraced to assess the methodological quality of the included studies (18). Two blinded reviewers (FDF, MF) independently made their assessment by reading the full-texts and a final discussion with a third reviewer (VC) resolved each discrepancy. This tool includes eight questions considering selection criteria, subjects and setting description, exposure and condition measurement, confounding factors identification and management, outcome measures, and statistical analysis. Assessors can give their response considering four possible answers: yes, no, not clear, not applicable.

### 2.4 Measures and synthesis of results

The primary outcome of this review was the difference in spatial-temporal parameters (e.g., velocity, cadence, stride length, step width, duration of gait cycle), and in trunk and lower limb kinematics (e.g., ROM in frontal, sagittal and transverse plane, motion patterns, coordination, variability and symmetry indexes) during walking between NS-LBP and healthy people.

As measurements of the kinematics parameters, we reported results and differences among groups in a descriptive way, by using mean ± standard deviation (SD) and mean and 95% confidence interval (CI). Results were considered statistically significant when the *p*-value was reported lower than 0.05.

The meta-analysis was performed using “Review Manager 5.4” (The Nordic Cochrane Center, https://training.cochrane.org/online-learning/core-software-cochrane-reviews/revman). We performed the meta-analysis only when at least two studies—comparable in terms of PECO parameters—investigated at least one of the considered outcomes. We considered the mean difference (MD) along with its 95% confidence interval (CI), calculated using a random-effects model. An effect size ranging from 0.2 to 0.49 is to be considered “small,” from 0.5 to 0.79 “moderate,” and if greater than 0.8, it is “large”. I2 statistics was calculated to measure heterogeneity, explaining how much the variations among studies are attributable to heterogeneity rather than chance. The interpretation of I2 values was realized as follows: 0%–40% “no importance”, range: 30%–60% “moderate”, range: 50%–90% “substantial”, and 75% or above “considerable” (19).

The quality of evidence was rated through the Grades of Recommendation, Assessment, Development and Evaluation (GRADE) method, as suggested by the Updated Cochrane Back Review Group method guidelines. This specific framework provides that it is possible to downgrade the quality of evidence from “high” to “moderate”, “low” or “very low” based on 5 key-domains: risk of bias, inconsistency, indirectness, imprecision, and publication bias (20).

## 3 Results

### 3.1 Study selection

Overall, the search strategy identified 1,466 results, 838 of which were duplicates and were consequently removed. An additional 600 records were excluded after screening for title and abstract. A total of 28 full-text articles were evaluated for eligibility and 9 studies were finally rejected with reasons (for further details see Figure 1). Additionally, 10 studies (9, 21–29) were also considered in the meta-analysis, the remaining 9 only in the qualitative summary (11, 30–37).

### 3.2 Description of the studies

All the included studies presented a cross-sectional design with at least one direct comparison between NS-LBP subjects and healthy people. In 5 studies (26%) the authors implemented three groups, by dividing their sample into specific classes (i.e., obese subjects, subjects presenting lumbar disc herniation, subjects with pronated feet, dancers, and subjects referring leg pain); however, the main comparison focused on the differences between NS-LBP and healthy subjects was always present. The aggregate number of participants was 725 and 410 of these were female (57%); the subjects were almost equally distributed across the studies (sample size median: 38, average: 38.15, IQR: 22–55, range: 20–70). The mean age of the participants was 36.33 ± 9.01 (median: 37, IQR: 32–41, range: 22.4–55.4).

Fourteen (74%) out of the 19 included studies reported data regarding pain intensity or disability levels at baseline. In particular, four studies (21%) only assessed pain intensity through a 0–10 or a 0–100 visual analog scale (VAS) or a 0–10 numeric rating scale (NRS); two studies (11%) investigated the functional status by using the “Oswestry disability index” (ODI) or the “Roland and Morris disability questionnaire” (RMDQ); eight studies (42%) assessed both of these outcomes. The mean pain intensity score of the NS-LBP population recruited in the studies was 4.14 ± 1.33 [range: 2.1–5.7], and the mean disability score was 24.37 ± 9.07 [range: 12.5–40.5].

Seventeen studies (89%) assessed kinematics through the optoelectronic motion analysis system, while two studies (11%) adopted wearable inertial measurement unit (IMU) sensors.

Further details are shown in Table 1.

### 3.3 Outcomes

All the included studies assessed at least one of the following walking kinematic indicators: spatial-temporal parameters, range of motion, qualitative indexes and motion patterns; each of these variables were considered as the primary outcomes in the current review.

In detail, twelve studies (63%) investigated spatial-temporal parameters; all of these considered the “gait speed” and the “cadence”, five (26%) assessed the “step length” and three (16%) measured the “stride length”. In this context, other considered indicators were step time, step width, single and double support time, and percentages of gait cycle.

Thoracic and lumbar kinematics was investigated in eight works (42%), whereas in eleven studies (58%) the lower limbs’ kinematics was measured for at least one of the following joint complexes: pelvis, hip, knee, and ankle.

Additionally, in eleven studies (58%) only quantitative parameters were retrieved, mostly peak angles and range of motion measurements. Conversely, six authors (26%) qualitatively assessed the walking kinematics by considering coordination, asymmetry, variability, and differences in motion patterns.

### 3.4 Methodological quality of the included studies

None of the included studies was completely judged free from methodological issues. Four (21%) out of the 19 included studies did not correctly state the inclusion criteria for their sample, and in the other two works (11%) this aspect was not clearly defined (risk of selection and reporting bias). In three studies (16%), we did not find any complete description regarding the sample characteristics or the research setting, and in five studies (26%) we judged this item as “not clear” (risk of selection and reporting bias). In addition, nine studies (47%) did not openly specify to have used objective and standard criteria for the participants’ selection, and in one case (5%) the procedure the authors adopted was judged as critical (risk of selection bias). Then, just one study (5%) identified all the potential confounding factors and only six studies (32%) used sample matching or multivariate analyses in order to manage some possible confounders. Finally, in two studies (11%) not enough details were provided regarding the outcome measurement (risk of detection bias) and in just one study (5%) an appropriate statistical analysis was not used, since only descriptive statistics were performed.

Further details are presented in Table 2.

**Table 2:** Methodological quality of the included studies assessed through the Joanna Briggs Institute (JBI) critical appraisal checklist for analytical cross-sectional studies.

First author (year)	1 Were the criteria for inclusion in the sample clearly defined?	2 Were the study subjects and the setting described in detail?	3 Was the exposure measured in a valid and reliable way?	4 Were objective, standard criteria used for measurement of the condition?	5 Were confounding factors identified?	6 Were strategies to deal with confounding factors stated?	7 Were the outcomes measured in a valid and reliable way?	8 Was appropriate statistical analysis used?
Al-Obaidi et al. (21)	No	NC	Yes	No	Yes	Yes	Yes	Yes
Amir Rashedi Bonab et al. (22)	Yes	Yes	Yes	Yes	No	No	Yes	Yes
Christe et al. (30)	No	No	Yes	NC	NC	Yes	Yes	Yes
Cimolin et al. (31)	Yes	Yes	Yes	Yes	No	No	Yes	Yes
Crosbie et al. (23)	NC	Yes	Yes	Yes	No	No	NC	Yes
Jiménez-Del-Barrio et al. (32)	Yes	Yes	Yes	NC	No	No	Yes	Yes
Ebrahimi et al. (33)	Yes	Yes	Yes	Yes	No	No	Yes	Yes
Farahpour et al. (34)	Yes	Yes	Yes	Yes	No	No	Yes	Yes
Gombatto et al. (23)	Yes	Yes	Yes	Yes	NC	Yes	Yes	Yes
Lee and Sung (24)	NC	Yes	Yes	Yes	No	No	Yes	Yes
MacRae et al. (25)	Yes	NC	Yes	Yes	NC	No	Yes	Yes
Müller et al. (9)	No	NC	Yes	NC	NC	Yes	Yes	Yes
Mullerpatan et al. (35)	No	NC	Yes	Yes	No	Yes	Yes	Yes
Nishi et al. (36)	Yes	No	Yes	Yes	No	No	Yes	Yes
Rahimi et al. (26)	Yes	Yes	Yes	Yes	No	No	Yes	Yes
Rum et al. (27)	NC	No	Yes	NC	No	No	Yes	Yes
Simmonds et al. (28)	Yes	Yes	Yes	Yes	NC	Yes	Yes	Yes
Simonet et al. (37)	Yes	Yes	Yes	Yes	NC	No	Yes	Yes
Vickers et al. (29)	Yes	Yes	Yes	NC	No	No	Yes	Yes

**Table 3:** Summary of findings.

Outcome	MD (95% CI)	N. of subjects (Studies)	Comments	Quality of Evidence
Gait speed	−15.42 (−22.78, −8.06)	189 (10)	Study design: CS Risk of bias: serious Inconsistency: serious Indirectness: serious Imprecision: serious^a^	⊕⊖⊖⊖ Very low
Cadence	−9.85 (−18.72, −0.99)	119 (5)	Study design: CS Risk of bias: serious Inconsistency: serious Indirectness: serious Imprecision: serious^a^	⊕⊖⊖⊖ Very Low
Step length	−6.30 (−11.83; −0.77)	105 (5)	Study design: CS Risk of bias: serious Inconsistency: not serious Indirectness: serious Imprecision: serious^a^	⊕⊖⊖⊖ Very Low
Stride length	−1.67 (−7.13; −3.78)	53 (3)	Study design: CS Risk of bias: serious Inconsistency: not serious Indirectness: serious Imprecision: serious^a^^,^^b^	⊕⊖⊖⊖ Very Low

CS, cross-sectional.

**Imprecision**:

a Small sample size.

b Wide confidence interval.

GRADE criteria.

**High Quality**: We are very confident that the true effect lies close to that of the estimate of the effect.

**Moderate quality**: We are moderately confident in the effect estimate; the true effect is likely to be close to the estimate of effect, but there is a possibility. that it is substantially different.

**Low quality**: Our confidence in the effect estimate is limited; the true effect may be substantially different from the estimate of the effect.

**Very low quality**: We have very little confidence in the effect estimate; the true effect is likely to be substantially different from the estimate of effect.

### 3.5 Description of results

#### 3.5.1 Spatial-temporal parameters

**Figure 2:**
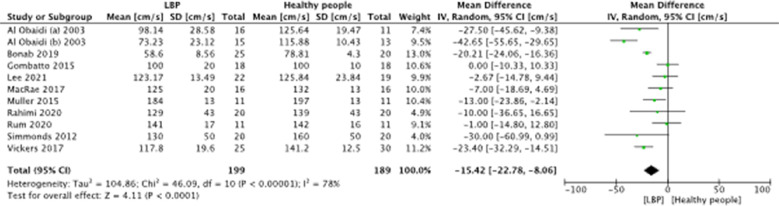
Forest plot of comparison between LBP subjects and healthy people. Gait speed (cm/s).

**Figure 3:**
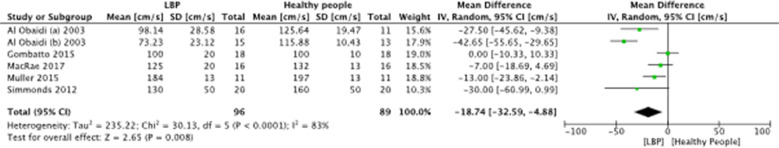
Forest plot of comparison between LBP subjects and healthy people (sensitivity analysis). Gait speed (cm/s.).

**Figure 4:**
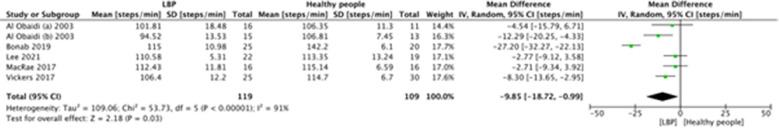
Forest plot of comparison between LBP subjects and healthy people. Cadence (steps/min).

**Figure 5:**
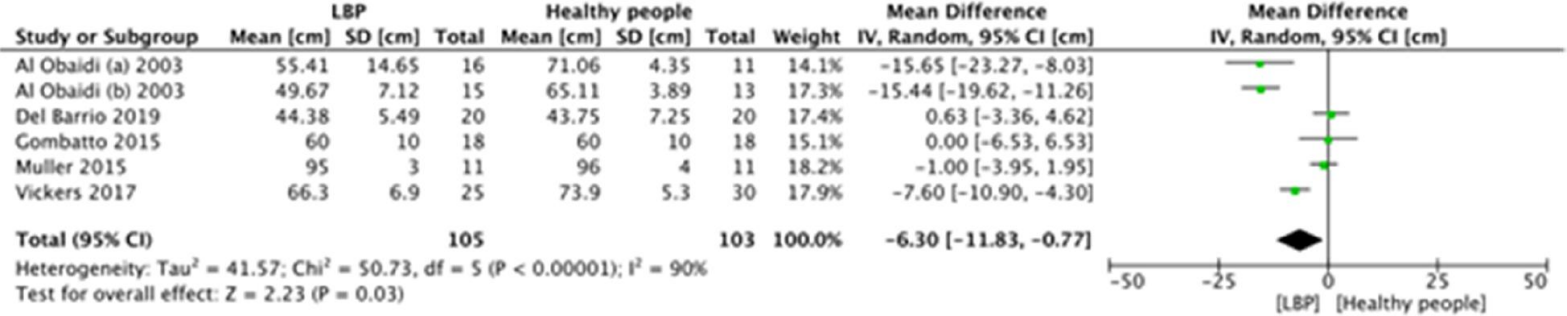
Forest plot of comparison between LBP subjects and healthy people. Step length (cm).

**Figure 6:**
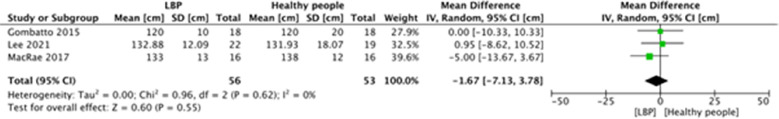
Forest plot of comparison between LBP subjects and healthy people. Stride length (cm).

A meta-analysis was possible for the following spatial-temporal parameters: gait speed, cadence, step length, and stride length; the corresponding forest plots are detailed in Figures 2–6. The analyses showed how people with LBP present a lower value of gait velocity with respect to healthy subjects [−15.42 (−22.78, −8.06) cm/s; *p* ≤ 0.0001], a lower value of cadence [−9.85 (−18.72, −0.99) steps/min; *p* = 0.03] and a lower value of step length [−6.30 (−11.83; −0.77) cm; *p* = 0.03]. No significant difference was observed in stride length [ −1.67 (−7.13; 3.78) cm; *p* = 0.55]. A sensitivity analysis (Figure 3) was performed for gait speed by excluding studies that had not controlled confounding factors, showing similar results to the general comparison [−18.74 (−32.59, −4.88) cm/s; *p* = 0.008]. The quality of evidence for each of these parameters was rated as “very low”. Further details are reported in Table 3.

Other parameters such as gait cycle time, single and double support time (stance and swing duration) and step width were measured occasionally and in different modalities (in seconds or in percentage of gait cycle), thus a quantitative summary was not possible. To summarize, in two studies (11%) a significant reduction in the single support duration was found in LBP people, only one of these (22) registered a decrease in step time and in gait cycle duration. In the same way, Cimolin et al. (31) detected a longer stance duration and a shorter step length in a population of obese LBP.

Other five studies (26%) assessed gait parameters without finding any significant difference with healthy people; among those, only Lee et al. (25) and Vickers et al. (29) measured step width, and no differences were observed as well.

#### 3.5.2 Quantitative kinematic parameters

As regards motion, a wide heterogeneity in the outcome measures was retrieved and a meta-analysis was not possible. Most of the authors (11 studies, 58%) investigated the range of motion (ROM) on the three different planes, by assessing the total range, minimum or maximum angles, or the difference in the peak angles during specific gait phases. Seven studies (37%) considered these endpoints on the lumbar or the thorax segments of the spine. Christe et al. found an overall less frontal-plane motion at the lower-lumbar segment (*p* ≤ 0.05) and a more asymmetrical motion in the transverse plane (30), while Gombatto et al. reported less overall rotation (*p* ≤ 0.05) (23). Conversely, Rum's study found a significant increase in the lumbar motion on the transverse plane (*p* ≤ 0.05) (27). Mullerpatan et al. reported a 20% increased spine extension in a population of NS-LBP dancers (35), while Simonet et al. pointed out a lower lumbar lordosis angle if compared to healthy subjects (hypotheses not tested by the authors) (37).

Eight studies (42%) assessed the lower limb kinematics, including pelvis, hip, knee, and ankle joints. Among those, two authors found an increased anterior pelvic tilt (*p* < 0.01) in NS-LBP subjects (32, 35), Muller et al. identified a decrease in pelvis rotation (*p* ≤ 0.05) (9), while Crosbie detected a smaller pelvic sidebending (*p* ≤ 0.05) (11). Mullerpatan et al. assessed hip ROM finding greater overall hip flexion (40%) (35), whereas Jiménez-Del-Barrio focused on a reduction in the hip extension (*p* < 0.01) (32). Farahpour et al. reported significant differences in peak angles in ankle inversion and eversion, and in knee flexion and external rotation (*p* ≤ 0.05) in a sample of NS-LBP with pronated-feet (34). Muller found a major knee extension at touchdown in NS-LBP people (*p* ≤ 0.05) (9). Cimolin et al. examined obese NS-LBP subjects, reporting an overall lower pelvis and hip ROM on the frontal plane, a lower knee flexion in the swing phase and a low ankle dorsiflexion in stance and swing, as compared to non-LBP obese people (*p* ≤ 0.05) (31).

Two studies (11%) did not find any significant difference in the lower limb kinematics (11, 25).

#### 3.5.3 Qualitative parameters for motion assessment

Six studies (32%) qualitatively assessed kinematics, by using different outcome measures; two of those assessed inter-segmental coordination. In detail, Crosbie et al. reported that “*phase lag*” for axial rotation was significantly less in subjects with LBP than controls at a preferred speed (*p* ≤ 0.01) and that lower thoracic regions displayed greater coherence (*p* < 0.001); the same author also calculated the *harmonicity index (IH)*, obtaining differences between groups for axial rotation in the lower thoracic and lumbar regions and for side flexion in the lumbar region, with subjects with LBP displaying greater IH values (*p* ≤ 0.05) (11). In the same way, Ebrahimi et al. found that LBP subjects demonstrated a less variable pelvis-thigh coordination pattern (*p* ≤ 0.05) (33); in addition, in LBP subjects the thigh-shank and shank-foot “*mean absolute relative phase*” (MARP) values were more in-phase than *MARP* values in healthy people during the swing phase (*p* ≤ 0.05).

One study (5%) considered the “*kinematic similarity index*” to assess the lower limb motion asymmetry during gait, finding lower values of similarities in the swing and stance phases in LBP people (*p* = 0.001). Two studies (11%) reported a greater trunk variability in LBP; in particular, Rum et al. reported an overall increase in the transverse plane (*p* ≤ 0.05) (27) and Nishi et al. obtained significantly higher values of “average standard deviation” of the antero-posterior and medio-lateral trunk accelerations (*p* ≤ 0.05) (36). Finally, Rahimi et al. assessed motion patterns, reporting significantly different hip motion patterns in the transverse plane, altered knee and ankle motion patterns in the sagittal plane, and different hip motion patterns in the transverse and frontal planes over the stance phase in the LBP group (*p* ≤ 0.05) (26).

## 4 Discussion

This systematic review with meta-analysis is, to our knowledge, the first to investigate gait kinematics in subjects with NS-LBP. In order to present the findings more clearly, we grouped them into two main categories: spatial-temporal parameters and quantitative kinematics.

When compared with healthy individuals, people with NS-LBP consistently showed alterations in the spatial-temporal domain, mainly walking at slower speeds, with reduced cadence and shorter step length. These results were confirmed by sensitivity analysis, where the exclusion of studies not controlling for confounders still supported a reduction in gait speed. Other parameters were less frequently reported, but there are consistent hints of reduced step time and gait cycle duration, findings that seem to extend also to obese NS-LBP subgroups. This suggests that temporal adaptations during gait may be a robust feature of this condition.

In the field of quantitative motion analysis, heterogeneity across outcome measures remains a limiting factor, preventing firm generalizations. Nevertheless, several studies converge in reporting lumbar and pelvic kinematic alterations during walking, especially in the transverse and frontal planes, with additional changes also observed at hip and knee levels. Obese and pronated-feet NS-LBP individuals also displayed ankle motion modifications. Furthermore, gait patterns in NS-LBP appear less coordinated, more asymmetric, and more variable than those of healthy subjects, even though these findings were not always consistent across the literature.

Although some studies did not detect substantial differences (23–28), the majority of available evidence points to altered spatial-temporal parameters in NS-LBP, probably driven by shorter step length and reduced cadence (21). Pain could be a primary driver of these adaptations, as there is evidence that individuals with LBP often take shorter steps on the affected side (38–40), and that pain intensity correlates with temporal gait parameters (22). However, non-physical factors may also play a relevant role, as highlighted by studies reporting the influence of fear-avoidance beliefs, anticipation of pain, anxiety, depression, awareness of being observed, and disability levels (29, 41–45). These results underline the multifactorial nature of gait adaptations in NS-LBP and suggest that future observational research should better disentangle the physical and psycho-behavioral contributors, as well as their interaction.

As discussed above, the most consistent quantitative kinematic finding is the reduction in lumbar spine ROM, particularly in the transverse and frontal planes. This has also been confirmed by studies excluded from our review for methodological reasons (14, 46, 47). The extent of this restriction varies across studies, but focusing on axial rotation, the reported loss ranges between one and four degrees. Such values are biomechanically meaningful (23), since reduced lumbar mobility can compromise dynamic stability and overall gait efficiency. One possible explanation is that NS-LBP subjects stiffen their lower lumbar segments during functional activities such as walking (48). Abnormal EMG activity of the extensor muscles may also contribute (49). Notably, both Christe et al. and Gombatto et al. reported greater restriction in the lower lumbar spine compared with the upper, consistent with the more frequent localization of pain in these segments (23, 30). However, contradictory findings were also observed: Mullerpatan et al. described increased lumbar extension (35), while Simonet et al. reported a lower lordosis angle (37). These discrepancies may reflect specific sample characteristics; for example, Mullerpatan's study involved dancers with LBP, a population known for hyperextension patterns, higher risk of spondylolysis (50), and motor control issues (51).

Regarding lower limb kinematics, results were highly inconsistent, likely due to the heterogeneity of sub-populations (e.g., obese, pronated feet, professional dancers). Nonetheless, decreased pelvic tilt, altered hip and knee excursions, and reduced inter-segmental coordination emerged as recurring patterns, often accompanied by greater asymmetry and variability. These findings may be related to muscle imbalances (52), as weakness, shortening, or elongation of certain muscle groups can affect joint motion and lead to compensatory kinematic changes (53, 54). It remains debated whether such alterations are direct consequences of pain or the result of long-term adaptations over time (55, 56). Clinicians should therefore adopt a comprehensive physical assessment when dealing with NS-LBP patients, considering both segmental deficits and global functional interactions.

Concerning the quality of evidence, only the meta-analyzed outcomes (gait speed, cadence, step length, and stride length) were assessed. All were rated as “very low” because of the observational design, inconsistency, indirectness, and imprecision. Specifically, heterogeneity was evident in gait speed, cadence, and step length, likely due to sample differences in age, BMI, pain intensity, and disability levels, as only a minority of studies matched participants for confounders. Consequently, downgrading for inconsistency and indirectness was required. Finally, sample sizes were generally small (below 400), and for stride length the confidence interval crossed the line of no effect, justifying further downgrading for imprecision.

Taken together, the findings suggest that NS-LBP patients display measurable modifications in both spatial-temporal and lumbo-pelvic kinematics during gait. These changes highlight the need for careful clinical assessment (57) and open the possibility for targeted therapeutic interventions. Specific training programs may help restore mobility and coordination (58), with gait assessment serving as a valuable tool to monitor progress.

Our findings complement previous meta-analyses addressing spinal kinematics across multiple tasks in LBP (59), by narrowing the scope to gait in non-specific LBP and providing a quantitative synthesis of spatio-temporal parameters. This focused approach refines the clinical interpretation of lumbopelvic motion during walking and its implications for assessment and rehabilitation.

Future studies should continue along this research line, both from a technological and clinical perspective. On the technological side, greater efforts should be made to validate and apply wearable solutions, such as IMU-based systems, which could represent a feasible alternative to marker-based optoelectronic devices still predominant in the field. On the clinical side, investigations should focus on clarifying the relative contributions of pain, physical adaptations, and psycho-behavioral factors (e.g., fear-avoidance, anxiety, avoidance strategies) (55). Additionally, more rigorous study designs are needed, including matching for confounders or focusing on specific subgroups of NS-LBP patients, to increase the robustness of findings.

Finally, the relevance of objective motion analysis in NS-LBP should also be acknowledged at the healthcare policy level, to ensure broader access and integration of these methods in clinical practice.

As with any study, this review has limitations. Despite a blinded and comprehensive search strategy, the breadth of the field may have led to some omissions. Moreover, the wide variability of kinematic parameters across studies prevented us from conducting meta-analyses in several domains, resulting in a qualitative synthesis with limited generalizability. As in all systematic reviews, publication bias remains a possibility and cannot be entirely ruled out (60).

## 5 Conclusions

There is very-low-quality evidence indicating that individuals with NS-LBP walk with reduced gait speed, cadence, and step length, while stride length appears comparable to healthy controls. A decrease in lower lumbar and pelvic motion, particularly in the transverse and frontal planes, is also observed.

Further high-quality studies are needed to strengthen the evidence and clarify whether these kinematic alterations are primarily driven by pain, physical adaptations, or psycho-behavioral factors.

## Data Availability

The original contributions presented in the study are included in the article, further inquiries can be directed to the corresponding author.
